# Influence of Modified Epoxy Resins on Peroxide Curing, Mechanical Properties and Adhesion of SBR, NBR and XNBR to Silver Wires—Part II: Application of Carboxy-Containing Peroxy Oligomer (CPO)

**DOI:** 10.3390/ma14051285

**Published:** 2021-03-08

**Authors:** Joanna Chudzik, Dariusz M. Bieliński, Michael Bratychak, Yuriy Demchuk, Olena Astakhova, Marcin Jędrzejczyk, Grzegorz Celichowski

**Affiliations:** 1 Institute of Polymer & Dye Technology, Lodz University of Technology, 90-924 Lodz, Poland; joanna.chudzik@edu.p.lodz.pl; 2 Institute of Chemistry and Chemical Technologies, Lviv Polytechnic National University, 79-013 Lviv, Ukraine; mbratychak@gmail.com (M.B.); yuriy_demchuk@ukr.net (Y.D.); KhTNH.dept@lpnu.ua (O.A.); 3 Institute of General and Ecological Chemistry, Lodz University of Technology, 90-924 Lodz, Poland; marcin.jedrzejczyk@p.lodz.pl; 4 Department of Materials Technology and Chemistry, Faculty of Chemistry, University of Lodz, 90-936 Lodz, Poland; grzegorz.celichowski@chemia.uni.lodz.pl

**Keywords:** rubber, oligomeric peroxide, modification, crosslinking, adhesion

## Abstract

This research was aimed at verifying the effect of carboxy-containing peroxy oligomer (CPO) addition on the possibility of rubber crosslinking and a subsequent adhesion of the modified rubber to silver wires. Three commonly industrially used rubbers were selected for the study: styrene–butadiene rubber (SBR), acrylonitrile–butadiene rubber (NBR) and carboxylated acrylonitrile–butadiene rubber (XNBR), together with carboxy-containing peroxy oligomer (CPO). An improvement in the adhesion of rubbers to silver wires was observed when applying the oligomeric peroxide with functional groups, with no deterioration of mechanical properties of the vulcanizates. Crosslinking synergy between dicumyl peroxide (DCP) and the modifier could hardly be observed. Nevertheless, the studies demonstrated, that to a small extent, even the CPO itself can crosslink NBR and especially XNBR, resulting in a material of notable elasticity and adhesion to silver wires.

## 1. Introduction

The current state of knowledge on how to improve adhesion between polymer and metals was described in the first part of our work [1]. It was devoted to epoxy resin containing peroxide group (PO) [2], whereas the second part of the paper deals with carboxy-containing peroxy oligomer (CPO) [3]. The aim of the study was similar—to determine the effect, this time of CPO addition, on the mechanical properties of selected synthetic rubbers and their adhesion to silver. Silver wires were selected as a tester to check the pro-adhesive potential of the resin towards some important technical rubbers: styrene–butadiene (SBR), acrylonitrile–butadiene (NBR) and carboxylated acrylonitrile–butadiene (XNBR). Experiments performed with macroscopic silver wire allow verifying the ability of CPO resin to improve adhesion between silver nanowires (AgNW) and rubber matrix in future rubber nanocomposites. Apart from the effect on interphase interactions, an addition of the resin is likely to modify crosslinks density and the structure of peroxide-cured rubber vulcanizates [1]. As discussed in the first part of our publication, such modification can be additionally potentially beneficial in terms of the mechanical properties of rubber/AgNW nanocomposites, their adhesion and conducting behavior.

## 2. Materials and Methods

### 2.1. Materials

#### 2.1.1. Synthesis of an Oligomeric Modifier

**Scheme 1:**
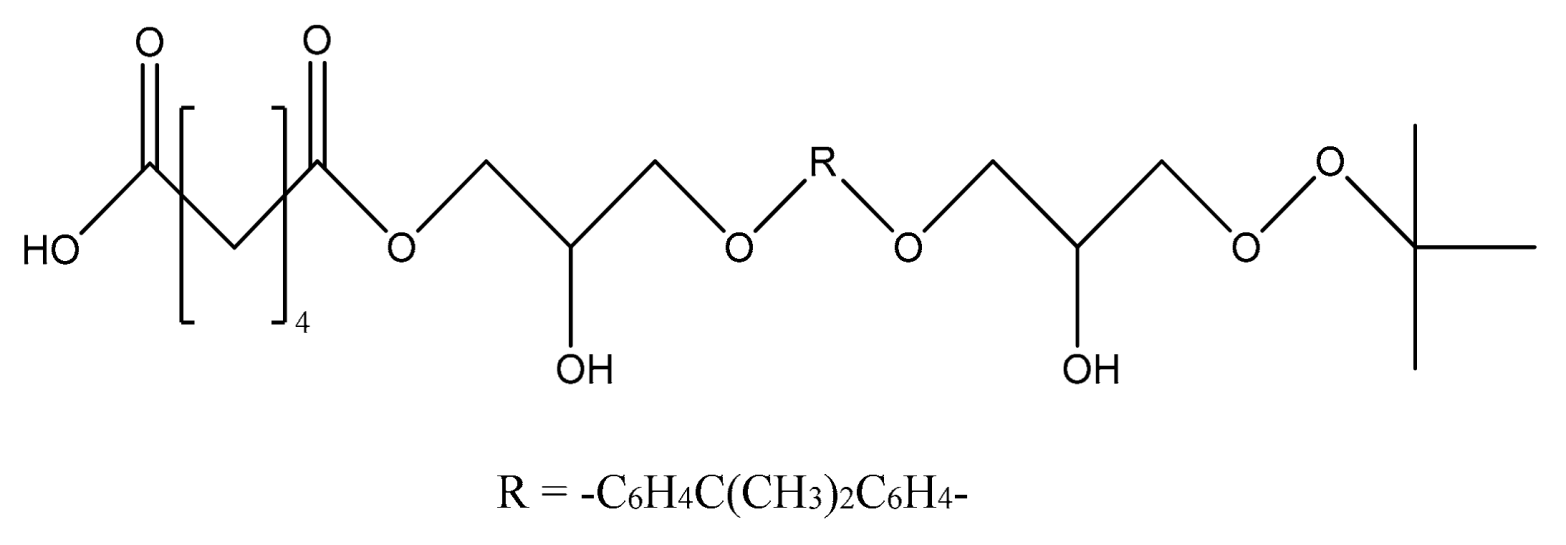
The chemical formula of CPO.

Carboxy-containing peroxy oligomer (CPO) of the formula presented in Scheme 1 was synthesized in a three-necked reactor equipped with a mechanical stirrer, reflux condenser and thermometer.

Thirty grams (30 g) of monoperoxy derivative of epoxy resin (PO) [2] dissolved in 150 mL of isopropanol, 3.7 g of benzyl triethylammonium chloride dissolved in 3.3 mL of water and 12.0 g of adipic acid dissolved in 50 mL of isopropanol were placed in the reactor. The reaction mass was sustained at 60 °C under stirring for 20 h. Then, 150 mL of toluene was added, and the mixture was carried into the dividing funnel. The bottom layer was not used, whereas the upper one was washed by water until all the catalyst was removed. The organic layer was then transferred to a vacuum distillation unit. Vacuum distillation was carried out at 50 °C and a residual pressure of 133–266 Pa to constant weight. In this way, 39.6 g of CPO oligomer of 560 g/mol molecular weight, with 2.0% of active oxygen and 8.8% of carboxyl groups content was obtained. The product did not have any epoxy groups. Their absence was confirmed by two methods: chemical and spectral. Chemical method: the epoxy number was determined using the back titration of hydrochloric acid acetone solution by 0.1 N alkali solution [3]. Spectral method: the absence of absorption bands at 910 cm**^−^**^1^ (typical of epoxy ring stretching vibrations) in the FTIR spectrum of CPO. Physical properties of the resin are presented in our previous paper [4].

#### 2.1.2. Preparation of Rubber Vulcanizates

Composition of the rubber compounds studied is presented in Table 1.

The rubber compounds studied were prepared in a Brabender Plasticorder laboratory micromixer (Germany), and then plate-calibrated to 2 mm thickness. The following mixing conditions were applied: rotational speed of 30 rpm; time of 7 min, room temperature. First, the rubber was plasticized for 3 min, then the appropriate amount of dicumyl peroxide (DCP, 98% of purity, Sigma-Aldrich Ltd., Poznan, Poland) was added and mixed together for another 2 min, and then the CPO modifier was added to the micromixer chamber. The whole content was then mixed for another 2 min. The samples were crosslinked in steel form under pressure, at 160 °C and during the optimum time of curing—t90, determined rheometrically, according to ISO 3417.

### 2.2. Methods

The research on the influence of CPO addition, on mechanical properties of selected synthetic rubbers and their adhesion to silver, was conducted in the same way as in the case of monoperoxy derivative of epoxy resin (PO). The experimental techniques applied have been described in the first part of the work [1]. Additional treatment with ammonia consisted of swelling the vulcanized samples in toluene under ammonia-saturated vapor in a desiccator at the room temperature for a period of 48 h (ν_A_) to recognize whether the crosslinked polymers contain any non-covalent crosslinks [5,6]. The concentration of the specific links was estimated from the difference between crosslink density determined by the equilibrium swelling of the vulcanizates in toluene (ν) and ν_A_. According to [7] and their own observations, NH_3_ contributes to the disintegration of the links formed at the filler–rubber interfaces.

## 3. Results

### 3.1. Kinetics of Crosslinking

**Figure 1:**
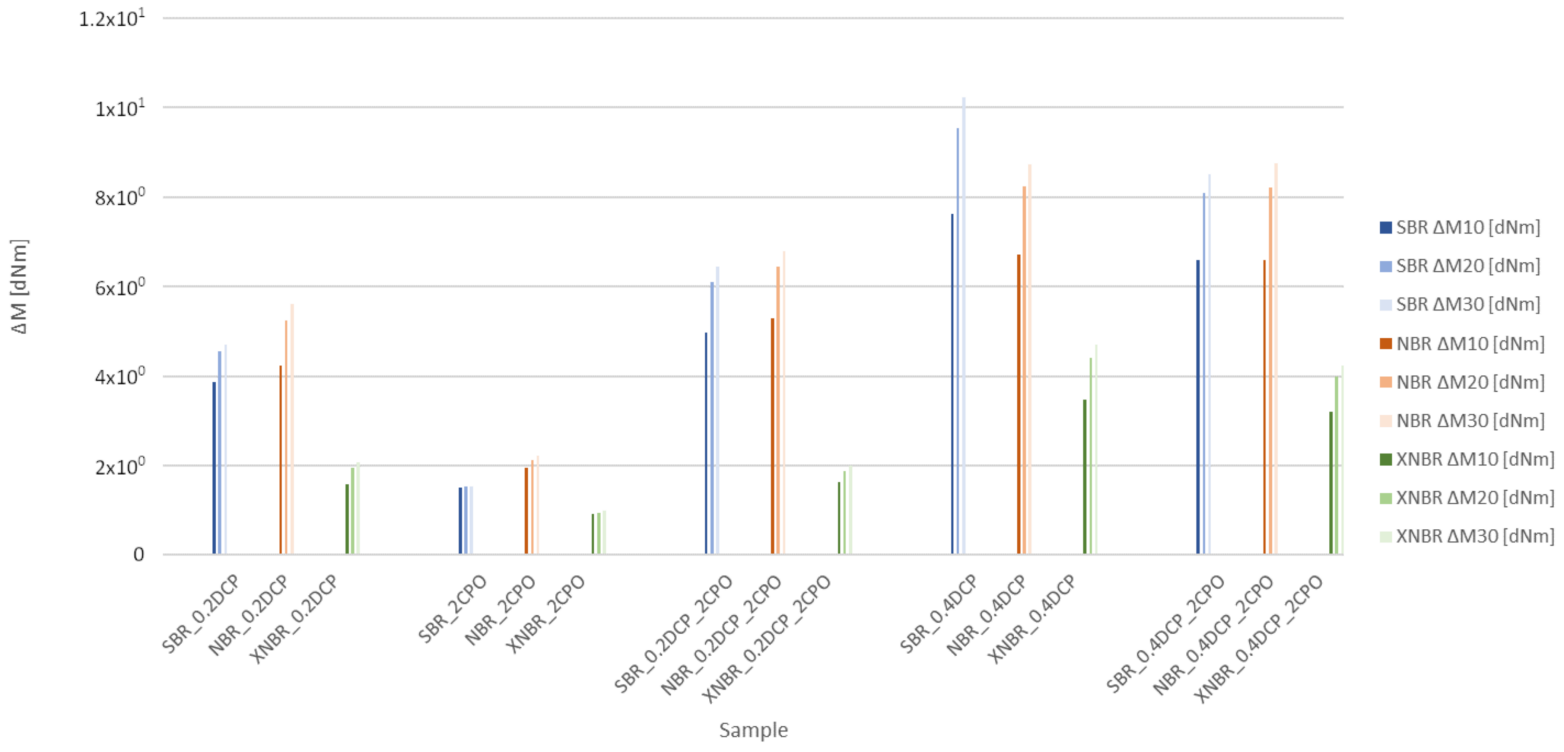
Vulcametric torque increase (ΔM) for the compounds studied at 10, 20 and 30 min from the beginning of vulcanization.

Crosslinking process of the rubber compounds tested is presented in Figure 1, while their conventional curing parameters are summarized in Table 2.

The analysis of the vulcametric data suggests the limited ability of the CPO oligomer to crosslink the tested rubber on its own. Adding CPO to XNBR had the smallest effect on torque increase, despite carboxyl groups being present in the rubber structure. However, the situation changes if CPO is used as a coagent of peroxide crosslinking in the presence of DCP. Initially, the effect expressed by an increase in the torque is most visible in the case of SBR-based compounds, whereas to a lesser extent for NBR-based mixes, but not present in the XNBR ones. ΔM was the largest if the modifier was added to a system containing 0.2 phr of DCP. For the vulcanizates crosslinked with a higher amount of peroxide (0.4 phr), CPO addition no longer made the vulcametric torque increased, adversely affecting ΔM value, which is especially visible for SBR and XNBR compounds. 

The addition of the resin practically does not influence either the optimum curing or scorch time of the compounds studied. There are no significant differences in the minimum torque values of the SBR and NBR rubber compounds tested, whereas for the mixes based on XNBR, it is clearly lower in comparison to the others.

### 3.2. Crosslink Density of Rubber

**Figure 2:**
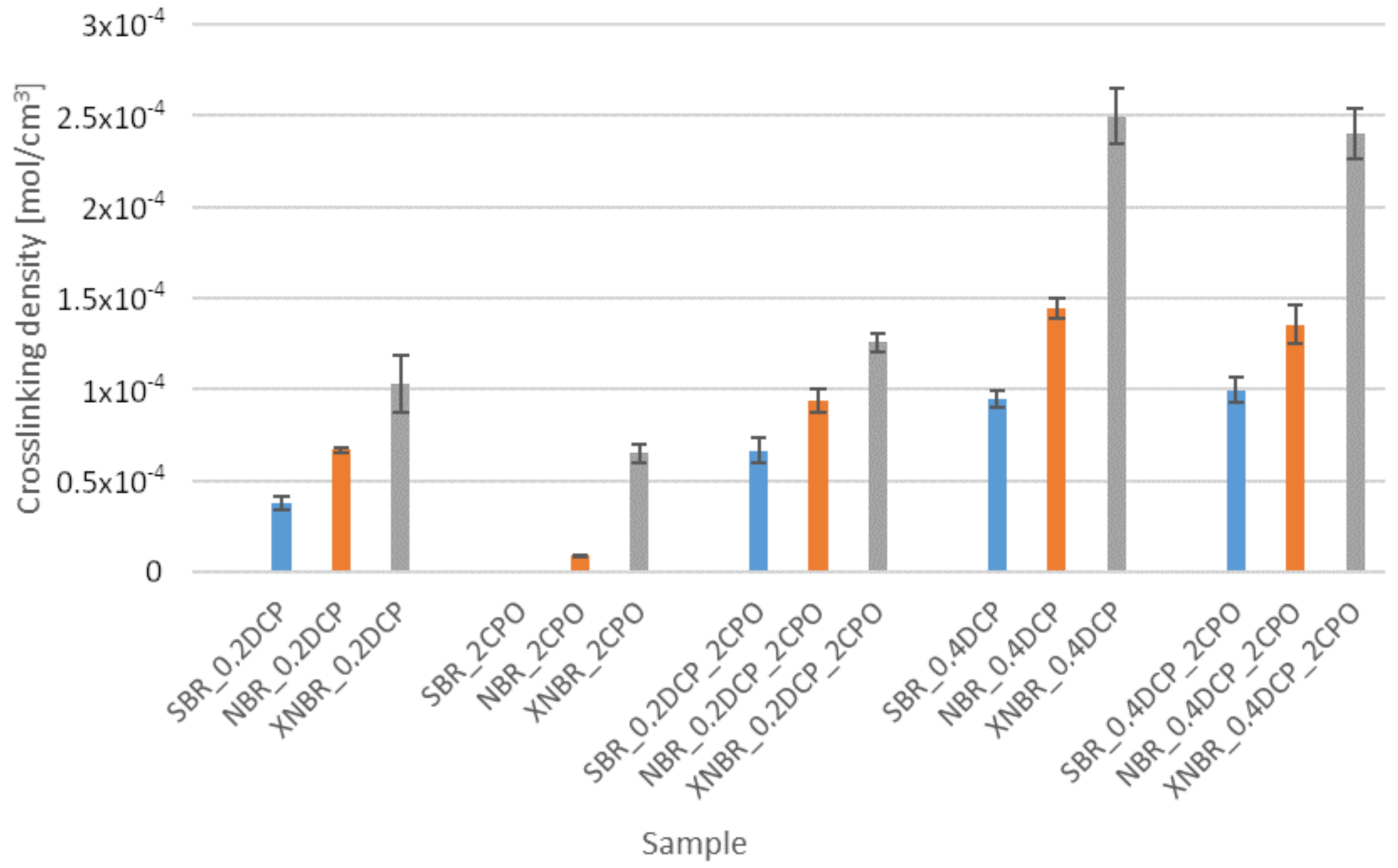
Influence of the carboxy-containing peroxy oligomer (CPO) addition on the crosslink density of the rubbers studied.

Comparison of the crosslink density of the cured rubbers tested, determined based on their equilibrium swelling in toluene, is presented for each of the rubbers in Figure 2.

Results of equilibrium swelling of the cured samples in toluene do not always match an increase in their vulcametric torque data. Nevertheless, the lower vulcametric torque of XNBR samples, despite their higher crosslink density in comparison to SBR and NBR ones, can be explained by the possibility of labile ionic crosslinks formation for the former. Measurements of vulcanizates’ equilibrium swelling in toluene and in toluene under ammonia vapors certified that carboxyl groups from CPO are involved in creating specific crosslinks in elastomer matrix. These interactions were destroyed by ammonia vapors—Table 3.

Only in the case of XNBR, rubber could be cured even without any addition of DCP. This may be due to the fact that CPO oligomer, like XNBR, contains carboxyl groups, thanks to which it has a good affinity to the rubber, which should be reflected by the strengthening and overall improvement of the physical properties of the rubber samples.

Compared to SBR, both nitrile rubbers achieve higher crosslink densities using DCP, because, unlike the former, they are additionally capable of thermal crosslinking (NBR) [8], or specific interactions of hydrogen bonds or ionic clusters formation (XNBR) [9,10]. A synergy of crosslinking action of the oligomeric modifier and DCP was confirmed for NBR and XNBR, but only when crosslinked using a 0.2 phr of DCP. The increase in DCP content causes a disappearance of the observed effect, which can most likely be subscribed to the total consumption of active macromolecular centers in the radical peroxide crosslinking reactions for SBR and NBR. In the case of XNBR, the formation of longer, labile ionic crosslinks can hamper the creation of covalent bond interactions [11].

The suggested mechanisms of rubber crosslinking with DCP was already presented elsewhere [12] and discussed in [1].
(1)
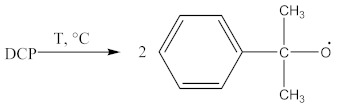

(2)
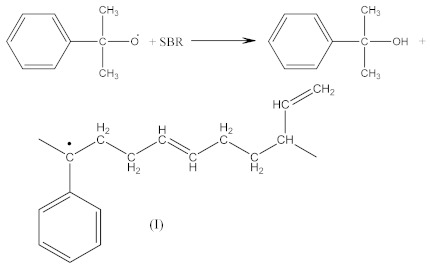

(3)
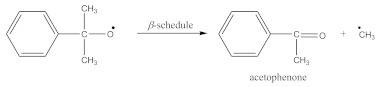

(4)
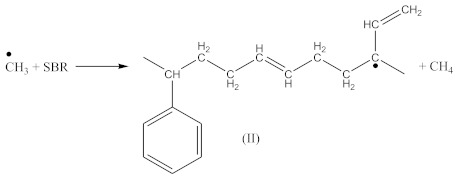

(5)
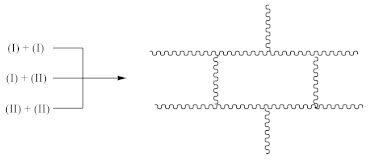


Crosslinking of SBR using CPO:
(6)
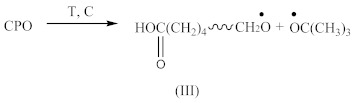

(7)
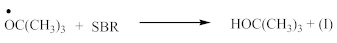

(8)
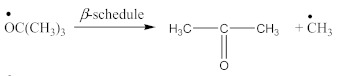

(9)
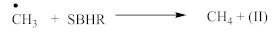

(10)
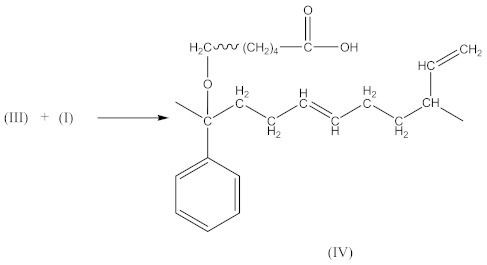


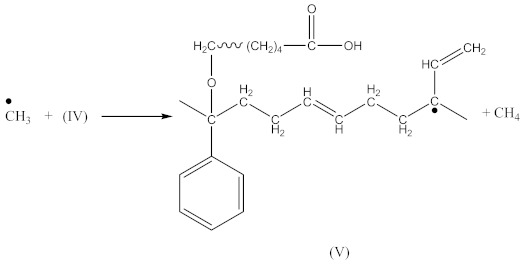
(11)
(12)
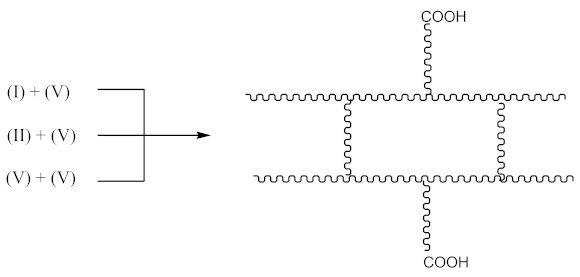



**Figure 3:**
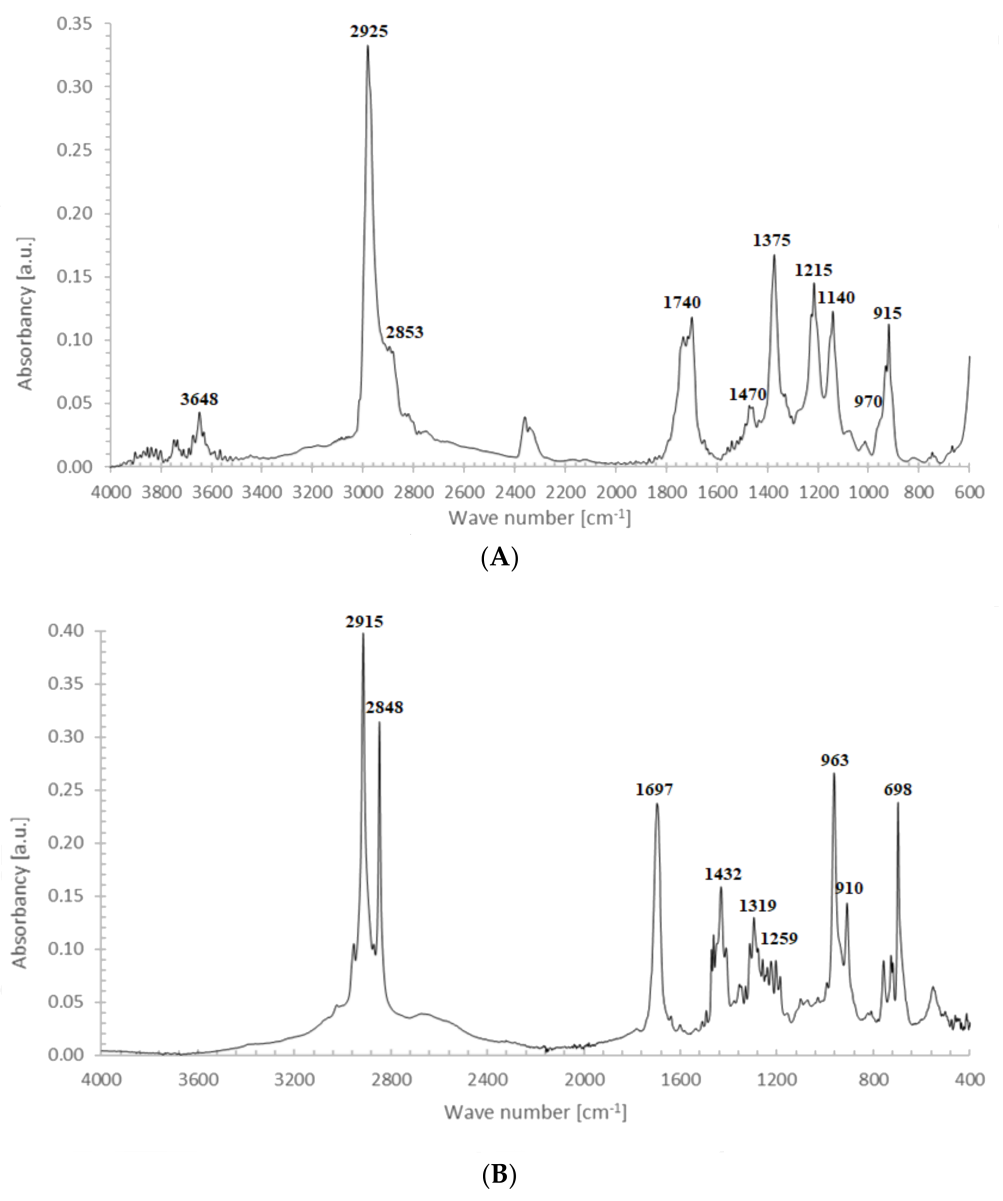
FTIR spectrum of SBR crosslinked with CPO oligomer: (**A**) CPO; and (**B**) SBR_2CPO.

FTIR spectrum of CPO (Figure 3A) contains the following characteristic absorption peaks:

- At 3648 cm^−1^, vibrations of free OH groups;
- At 2925 and 2853 cm^−1^, symmetric and asymmetric vibrations of CH_2_ and CH_3_ groups;
- At broad absorption at 1740 cm^−1^, related to C=O vibrations, probably from aldehyde groups;
- At 1470 and 1375 cm^−1^, stretching vibrations of CH_2_ and CH_3_ groups; and
- At 1325, 1215, 1140 and 1050 cm^−1^, vibrations in alcohol and/or ether fragments.

The peak at 1740 cm^−1^ significantly increases due to the thermal decomposition of CPO (6), also indicating on -COOH groups present in the spectrum of crosslinked rubber products (Figure 3B). Additionally, arising from the characteristic absorption peaks at 970 and 915 cm^−1^, coming from C–H bending vibrations in double pendant bonds, confirming on the proposed mechanism of SBR crosslinking with CPO (Equations (10) and (11)). 

In the case of SBR_2CPO cured at 160 °C (Figure 3B), the presence of free carboxy groups is proved by absorption bands at 1432 and 1697 cm^−1^, corresponding to the stretching vibrations of hydroxy groups and the carbonyl group in acid, respectively. The ester group is confirmed by the presence of bands at 1312 and 1259 cm^−1^, corresponding to the stretching vibrations of CO in saturated esters. The secondary hydroxy groups are proved by the band at 1050 cm^−1^. The absorption band at 1100 cm^−1^ corresponding to –C–O–C– asymmetric vibrations in ethers indicates the CPO molecule attachment to SBR. The presence of SBR molecules is confirmed by the absorption bands at 698, 910, 963, 2848 and 2915 cm^−1^, corresponding to C–H deformation vibrations in styrene, nonplanar deformation vibrations of C–H in –C=C–H, symmetric and asymmetric stretching vibrations of -CH_2_- in saturated hydrocarbons, respectively. Analogously to NBR, the attachment of CPO fragments to SBR occurs according to Equation (10), due to a recombination of radicals formed according to Equations (6), (7) and (9), preserving the unsaturated double bonds in the SBR molecule.

Crosslinking of SBR using DCP + CPO proceeds according to Equations (1)–(12).

Acrylonitrile–butadiene rubber (NBR):

Crosslinking of NBR using DCP proceeds according to Equations (1)–(4) [1].

The radical formed by Equation (2) has the form presented in Equation (13):
(13)
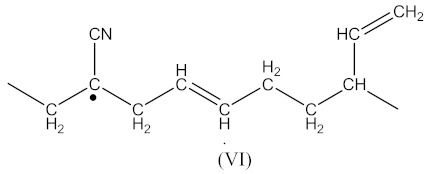

whereas the radical formed by Equation (4) has the form presented in Equation (14):
(14)
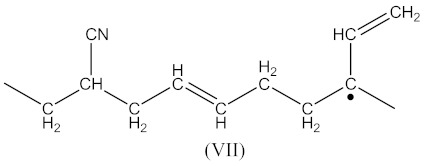


During curing by DCP, crosslinking occurs as a result of the interaction:

(15)
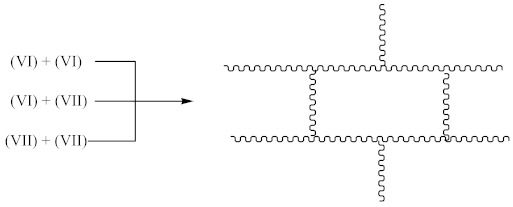


**Figure 4:**
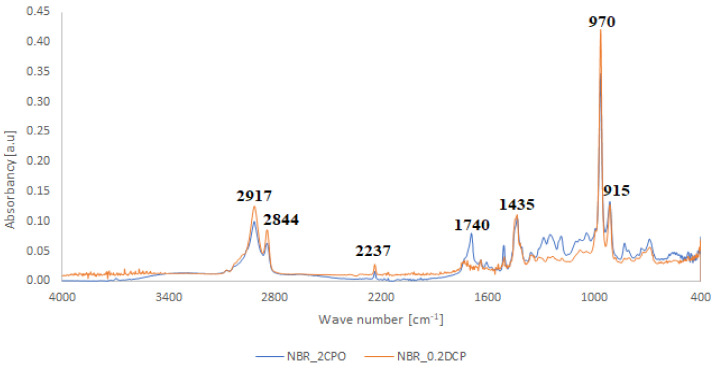
Comparison between the FTIR spectra of NBR crosslinked with CPO oligomer (NBR_2CPO) and DCP (NBR_0.2DCP).

Contrary to crosslinking SBR, the broad absorption peak at 1740 cm^−1^, indicating on the thermal decomposition products of CPO (6–8), only decreases in the spectrum of crosslinked NBR (Figure 4). Characteristic absorption peaks, originating from C–H bending vibrations in double pendant bonds at 970 and 915 cm^−1^ arise, confirming the proposed mechanism of crosslinking NBR with the CPO modifier. Crosslinking of NBR using DCP + CPO proceeds via the reactions proposed by Equations (12) and (13).

Carboxy-containing peroxy oligomer (CPO) contains free carboxy, secondary hydroxy and ester bond in its structure. As a result of the NBR peroxy curing the decomposition of –O–O– bond in the CPO molecule occurs in accordance with Equation (13). The formed (CH_3_)_3_CO• radical attacks the NBR molecule with a radical formation and transforms into tert-butyl alcohol. Moreover, this radical is converted into acetone molecule via β-schedule and forms CH_3_• radical, which further attacks the NBR molecule, detaches hydrogen and turns into CH_4_. This reaction again leads to the formation of a radical on the NBR molecule. The created radicals may recombine between each other to cure the rubber molecule, or they may attach an oligomer radical containing free carboxy, secondary hydroxy and ester fragment. The latter leads to NBR modification and the appearance of carboxy and hydroxy groups in its structure. The mentioned groups should affect the mechanical and adhesive properties of the rubber. In the FTIR spectrum of NBR_2CPO (Figure 4), one can see absorption bands at 1435 and 1693 cm^−1^ corresponding to the stretching vibrations of hydroxy and carbonyl group in the acid, respectively. The presence of a ester group is confirmed by the band at 1249 cm^−1^, corresponding to the asymmetric stretching vibrations of –C–O–C– in esters and by the band at 1186 cm^−1^, corresponding to the stretching vibrations of C–O in aliphatic acid esters. The presence of the secondary hydroxy group is proved by the absorption band at 1043 cm^−1^ corresponding to the vibrations of –C–OH group. The absorption band at 1100 cm^−1^, corresponding to the asymmetric stretching vibrations of –C–O–C– in ethers, indicates the attachment of the CPO molecule to the NBR molecule. The groups and fragments characterizing NBR are confirmed in the spectrum by absorption bands at 912, 964, 2237, 2844 and 2917 cm^−1^ corresponding to nonplanar deformation vibrations of –CH in –C=C–H (RHC=CH_2_ fragment), nonplanar deformation vibrations of –CH in –C=C–H (RHC=CHR’-trans fragment), stretching vibrations of –C≡N, symmetric and asymmetric stretching vibrations of -CH2- in saturated hydrocarbons, respectively. The attachment of CPO fragments to NBR occurs via a radical mechanism, mostly without the participation of NBR double bonds. Bands at 912 and 964 cm^−1^ corresponding to the double bond confirm this fact.

Crosslinking of XNBR using DCP:

The reactions occurs similarly to Equations (1)–(4). Two possible macroradicals can be formed. Similarly to Equation (2) of the appearance (Equation (16)):
(16)
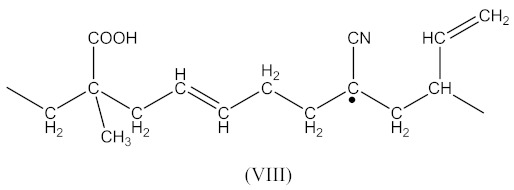

as well as similarly to Equation (4) in the form (Equation (17)):
(17)
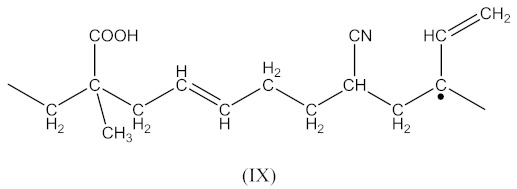


Then, the crosslinked structure is formed by the interaction (Equation (18)):
(18)
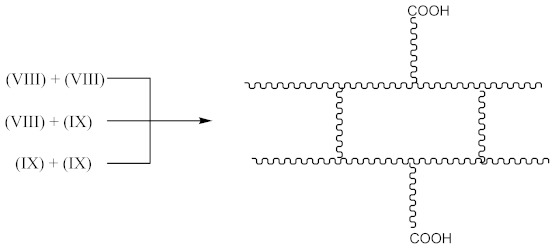


Crosslinking of XNBR using DCP and CPO:

In this case, two curing processes are imposed, separately in the presence of DCP according to Equations (1)–(4)—with the formation of radicals (II), and in the presence of CPO according to Equations (6)–(9)—with the formation of the compound (X) presented in Equation (19):
(19)
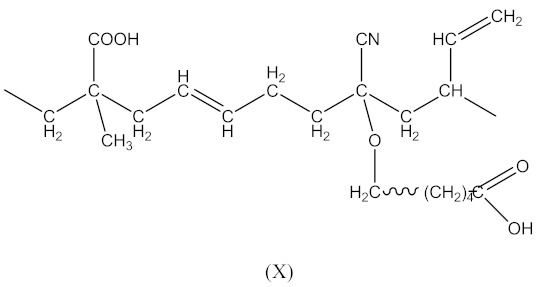

followed by the formation the macroradical (XI), similar to Equation (11) (Equation (20)):
(20)
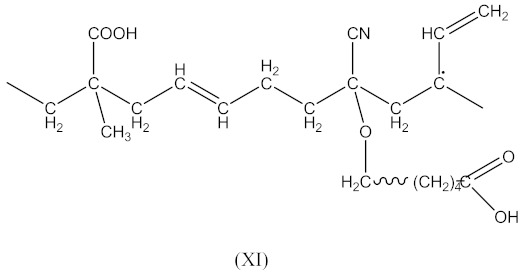


The subsequent recombination of the radicals leads to rubber crosslinking according to Equation (21):

(21)
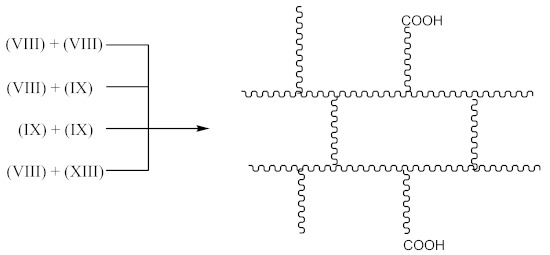


This in turn leads to an increase in the number of free carboxyl groups in crosslinked rubber and is probably responsible for specific interactions (non-covalent crosslinks) and the increase in adhesion to the metal.

In addition to the aforementioned mechanisms for the attachment of oligomeric radicals to rubbers and their subsequent crosslinking, an attack of the double-bonded rubber radical according to the scheme should also be considered:

(22)
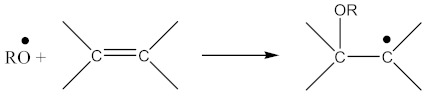


Radicals formed according to Equation (22) are capable of recombination according Equation (23):
(23)
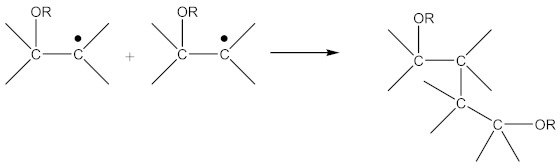

with the formation of a crosslinked structure. However, the reactions proceeding according to Equations (16) and (17) are limited to only 10–15% of all rubber crosslinked by peroxides [13]. 

**Figure 5:**
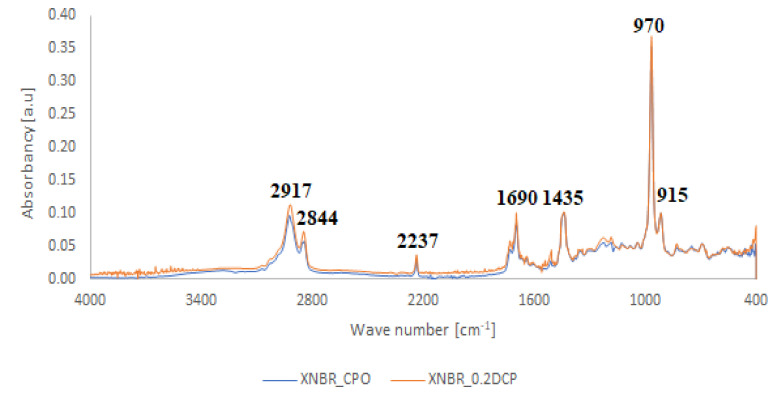
Comparison between FTIR spectra illustrating the way of crosslinking of XNBR with CPO oligomer (XNBR_2CPO) and DCP (XNBR_0.2DCP).

Contrary to the crosslinking of SBR and NBR with the carboxy-containing peroxy oligomer (CPO), in the FTIR spectrum of crosslinked XNBR (Figure 5), the absorption peak at 1740 cm^−1^, indicates that the thermal decomposition product of the oligomer (6–8), does not disappear for rubber crosslinked with CPO, but shifts to a lower wavelength 1680–1700 cm^−1^, indicating on their XNBR origin and probably covering the rubber crosslinking process. In the XNBR crosslinked with DCP, the carboxylic acid group should exist predominantly as a hydrogen-bonded acid dimer, which is represented by a characteristic infrared carbonyl stretching vibration at 1697 cm^−1^. Regarding the band associated with stretching vibration in the cyano groups at 2237 cm^−1^, the spectra show only slight differences in intensity, indicating small activity of these groups during crosslinking reactions [14]. Characteristic absorption peaks—originating from hydroxyl groups at 3200–3400 cm^−1^ and from C–H bending vibrations in the pendant double bonds at 970 cm^−1^ and 915 cm^−1^—arise in both cases, confirming the proposed mechanism of crosslinking XNBR with CPO oligomer.

### 3.3. Mechanical Properties of Rubber

**Figure 6:**
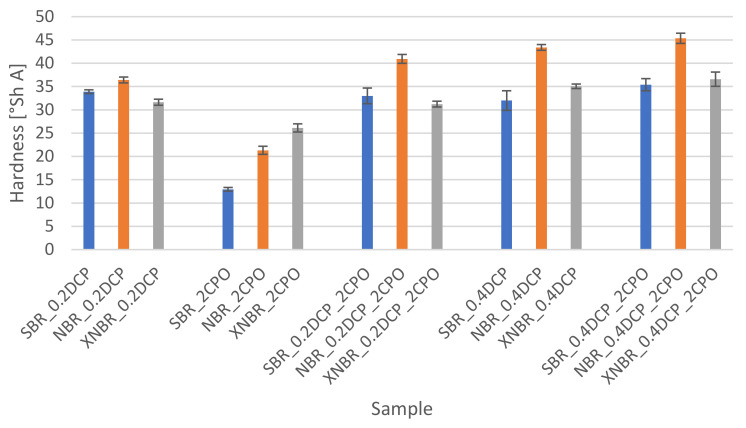
Influence of the peroxide curing system modification on the hardness of the rubber vulcanizates.

The mechanical properties of the crosslinked rubber samples are demonstrated in Table 4 and Table 5 and Figure 6.

#### 3.3.1. Tensile Strength (TS)

The influence of the crosslinking system on the mechanical strength of the crosslinked samples tested depends on the type of rubber. For SBR, there is a general trend that systems containing both an organic peroxide and an oligomeric peroxide with functional groups exhibit higher tensile strength than those based solely on DCP, whereas rubbers containing only CPO exhibit a much lower value of stress at a break in comparison to the others. Elongation at break values indicate that rubbers crosslinked with mixed systems (DCP/CPO) demonstrate a slightly higher strength but less flexibility than samples containing only with DCP. In contrast, rubbers crosslinked with CPO only exhibit much lower strength but are associated with higher elongation at break. Increasing DCP content results in an increase in the samples stiffness, but their tensile strength does not improve, due to significant reduction in elongation at break.

In NBR-based samples, there are no significant differences between stress at the break for mixed systems and systems based only on DCP. However, the former has the highest TS value. As in the case of samples based on SBR, the use of CPO oligomer alone as crosslinking agent significantly reduces the mechanical strength of the rubber with an increase in its elongation at break. In general, however, the mechanical parameters of NBR vulcanizates crosslinked with the mixed system are inferior to the corresponding SBR-based compounds.

In a group of XNBR-based samples, those containing only the oligomeric peroxide with functional groups (CPO) or 0.2 phr of DCP mixed with CPO, showed such high flexibility that they exceeded the measuring range of the testing instrument (no tearing), achieving in the case of the latter one of the highest stress values among the samples tested in this series. The use of mixed systems containing 0.4 phr DCP caused a decrease in specimen elongation at break with an increase in the corresponding stress.

#### 3.3.2. Tear Resistance (TES)

None of the samples crosslinked with a resin alone have been torn, similar to XNBR compounds crosslinked with 0.2 phr of DCP and the resin. For all the rubbers tested, it was observed that the addition of PO resin generally reduces the force needed to tear the sample. The exceptions are rubbers containing 0.4 phr of DCP and 2 phr of PO as a crosslinking system, for which the tear force increases. 

Therefore, it can be concluded that the introduction of the modified resins into the DCP crosslinking system improve the tear resistance of the crosslinked rubbers tested.

#### 3.3.3. Hardness

The impact of the modification applied to the peroxide crosslinking system on the hardness of the rubber samples tested is presented in Figure 6.

The addition of the oligomeric peroxide with functional groups (CPO) to the rubbers tested, especially in combination with the organic peroxide, causes a slight hardening of the samples, most evident in the case of those ones based on SBR. In turn, the use of the oligomer alone reduces rubber hardness, especially SBR and NBR, indicating on a plasticizing effect of CPO. In the systems based on XNBR, the observed changes to the rubber hardness, as a result of the modification of the crosslinking system composition, are the lowest among the rubber samples tested.

### 3.4. Adhesion

**Figure 7:**
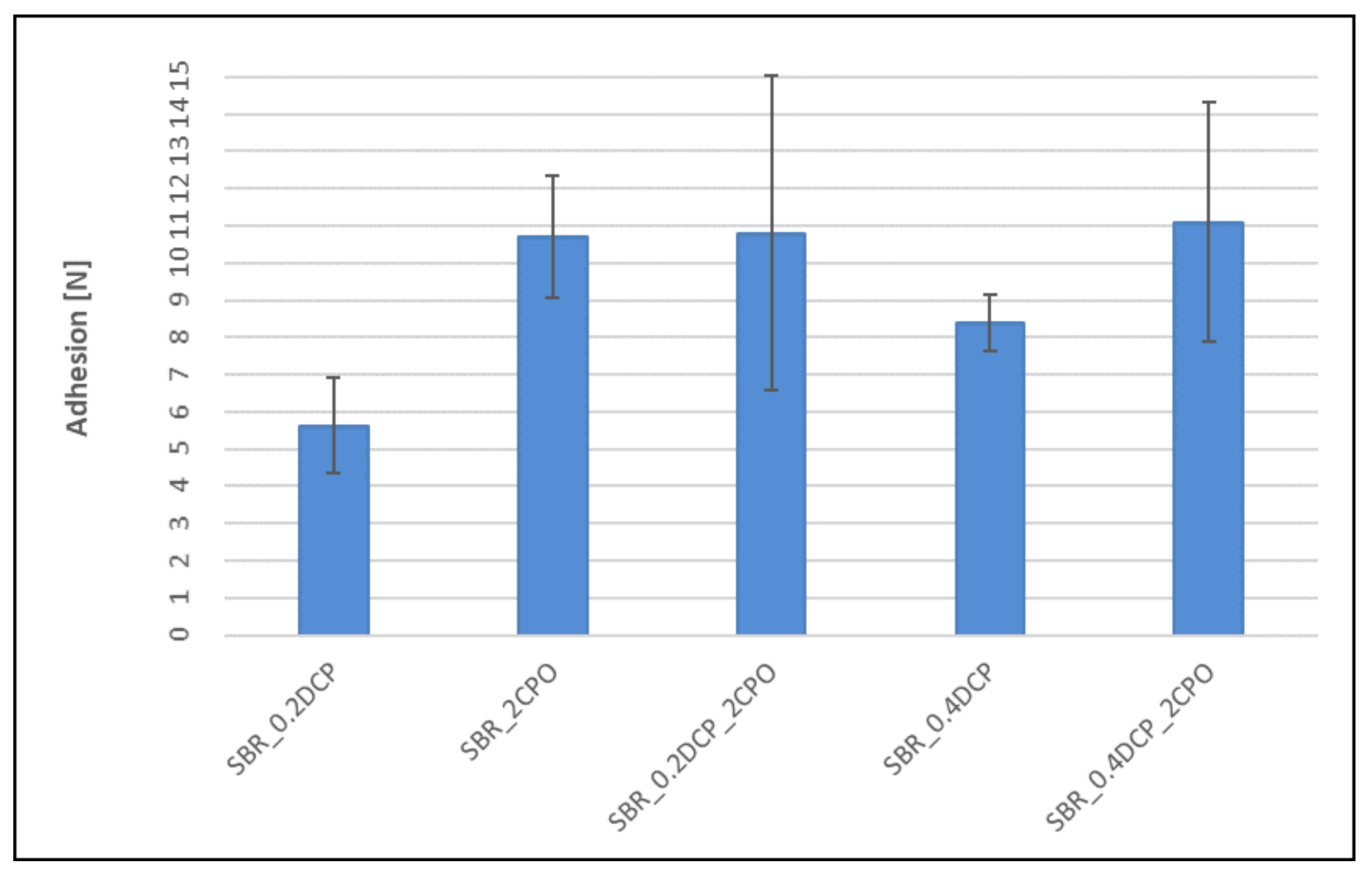
Influence of the peroxide curing system modification on adhesion between peroxide SBR vulcanizates and silver wires.

**Figure 8:**
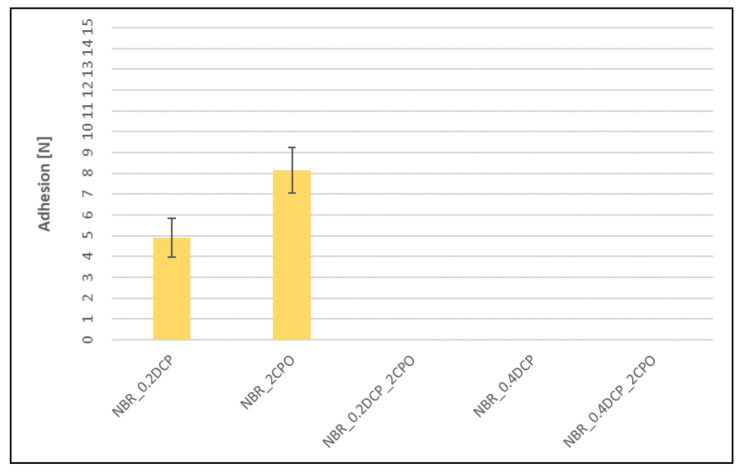
Influence of the peroxide curing system modification on adhesion between peroxide NBR vulcanizates and silver wires (missing bars indicate the lack of adhesion).

**Figure 9:**
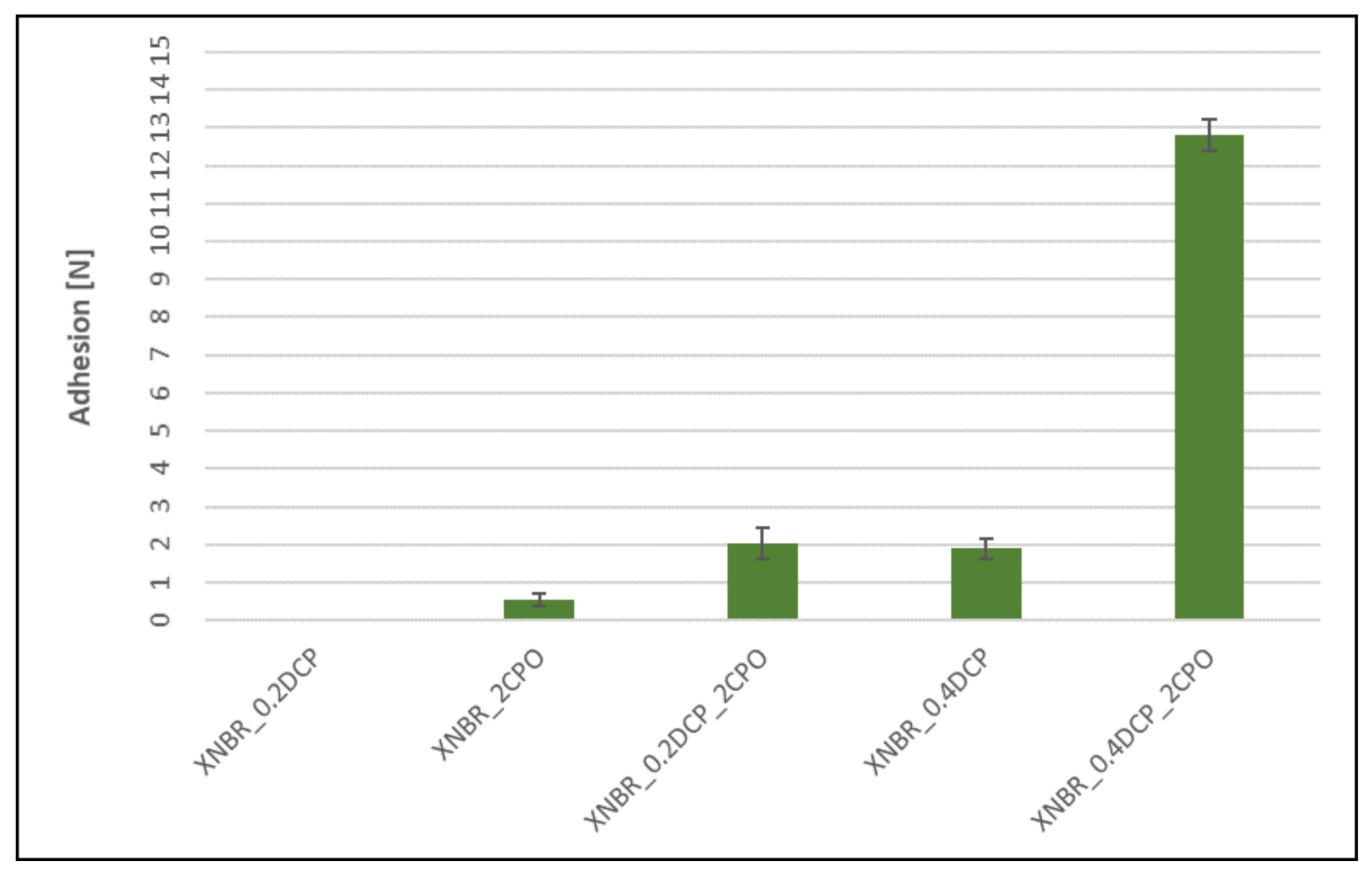
Influence of the peroxide curing system modification on adhesion between peroxide XNBR vulcanizates and silver wires (missing bars indicate the lack of adhesion).

Due to the inability of measuring the adhesion at the interface between the rubber matrix and the silver nanowires, a standard, the so-called “H” shape specimen method, often used for macroscopic wire/cord—rubber adhesion tests—was applied. Thus, information on the proadhesive potential of the oligomeric CPO additive in relation to selected synthetic rubbers crosslinked with dicumyl peroxide was expected. The results obtained are summarized in Figure 7, Figure 8 and Figure 9.

The addition of CPO to the peroxide rubber compounds, despite influencing crosslinking, increases adhesion between SBR-based vulcanizates and silver wires. As expected, the carboxyl group-containing peroxide oligomer significantly improves the adhesion. In each case, taking into account the mechanical strength of rubbers (Table 3 and Table 4), a cohesive destruction of the connection should be expected. Even the use of CPO alone (without DCP) results in a significant increase in adhesion compared to relatively high adhesion for a sample crosslinked with 0.2 phr of DCP. For NBR vulcanizates, no measurable adhesion is observed, neither for mixed systems (peroxide/CPO oligomer) nor for the sample crosslinked with 0.4 phr of DCP. On the other hand, the addition of CPO alone, without peroxide, strongly improves the adhesion to silver wires, just like for SBR-based samples. The matter of the adhesion of silver wires to the peroxide-cured XNBR looks similar to the SBR case, except that the improvement obtained is much smaller. The exception confirming the rule is a XNBR/0.4DCP/2CPO system, in which the measured adhesion force is the highest among all the crosslinked samples tested. This may be due to the fact that the CPO modifier, like XNBR, contains carboxyl groups, which give it a good affinity for the rubber, manifesting itself by a significant strengthening of the adhesion force and the overall improvement of the physical properties of the rubber samples.

## 4. Discussion

The ability of CPO for crosslinking selected synthetic rubbers of technical importance—SBR, NBR and XNBR—is limited. It has been known since 1940 that phenol-formaldehyde resins (resoles) or halomethyl phenols in the presence of selected metal chloride hydrates and an acid catalyst can be used, even without peroxides, for the crosslinking of unsaturated rubbers, such as: natural rubber (NR), styrene-butadiene rubber (SBR), ethylene-propylene-diene rubber (EPDM), butyl rubber (IIR) and acrylonitrile-butadiene rubber (NBR). However, it is worth mentioning that the curing time of acrylonitrile–butadiene rubber in this way is relatively low. Looking at the mechanism of such crosslinking in NBR, it can be noticed that amidine bonds are formed in it as the result of interactions with nitrile groups. The presence of the methyl group in CPO, which can be reactive to nitrile groups in the rubber, is not significant because the crosslinking agent is used in small quantity. Crosslinking with derivatives of epoxide resins may also be applicable to active hydrogen containing rubbers, such as Hypalon, acrylic rubber or XNBR [5]. Nevertheless, the crosslinking of rubbers with peroxide oligomers containing functional groups (like CPO) nor the properties of their vulcanizates have been studied. 

Despite the modifier only exhibits a limited synergistic effect on the peroxide (DCP) crosslinking of rubbers, the mixed systems containing DCP and CPO are more efficient than DCP or oligomeric peroxide added alone. This affects the mechanical properties of the cured rubbers, which can be also additionally modified by changes to crosslink structure, being a result of the introduction of ionic crosslinks and intermolecular specific interactions. The introduction of oligomeric peroxides with functional groups into the peroxide curing system does not cause drastic changes to the mechanical strength of rubbers studied; however, it can reduce their elongation at break and improve the tear strength of the samples. With a few exceptions, the addition of CPO has also no significant effect on the peroxide curing parameters of the rubbers tested.

The addition of the oligomeric peroxide also results in a significant increase in adhesion between the SBR rubber and the silver wire. This fact can be used to help the research devoted to nanocomposites filled with silver nanowires, which can be used as mechanical stress sensors [15]. Slightly smaller improvement in adhesion has been observed for XNBR, however, the rubber cured with a system containing 0.4 phr of DCP and 2 phr of CPO proves to be unique in this respect by demonstrating even higher adhesion than that determined for SBR vulcanizates. The studies showed that due to the lack of adhesion between the NBR rubber and the silver wire, it is not advisable to use this rubber as a matrix for stress sensors containing silver nanowires. Despite the fact that NBR cured with CPO alone (without DCP) causes a significant increase in its adhesion to silver wires, the mechanical properties of the vulcanizates disqualify any structural or functional applications.

The same silver wire was used and the same cleaning procedure was implemented in all experiments so, it can be assumed that the mechanical component of adhesion is comparable for all cases. This means that the chemical component of adhesion is responsible for different pull-out forces determined for various rubber vulcanizates. Indeed, hydroxyl groups present on the surface of oxidized silver [16] could react with the carboxylic group from CPO-modified rubbers, explaining an increased adhesion to silver.

In examined systems, adhesion has a mixed chemical–mechanical nature. The observed differences in the measured adhesion forces result mainly from the different chemical interactions on the polymer–metal interface. The modification of rubbers with CPO resin makes carboxyl groups attached to the end of macromolecular chains, as presented by the structures (V) and (X). This provides them with a better access to the silver surface than the carboxyl groups originally found in XNBR—directly attached to the main polymer chain, which is probably a significant steric hindrance, limiting the interactions between COOH groups and the metal surface. The presence of suitable reactive groups (COOH and OH) on both sides of the interface, together with the heat and pressure present during vulcanization, promote the chemical reaction leading to the formation of covalent or covalent–ionic bonds [17]. In the case of phenylic groups present in SBR, another important chemical process along metal–polymer interfaces can be disclosed and namely the interaction of 4d electrons from transition metals such as silver with aromatic rings (π-electron) in polymers resulting in the formation of π−4d electrons interaction [18]. The mechanism explains high adhesion between silver rods and rubber in case of SBR, containing aryl group, absent in the case of NBR or XNBR. 

Another parameter, in addition to the presence of functional groups, responsible for the modified rubber—silver adhesion is the degree of the rubber matrix crosslinking. A silver rod is easily pulled-out from a low crosslinked rubber, that residues remain attached to the metal surface. The strong chemical adhesion manifests itself for a highly crosslinked rubber, which explains the highest adhesion determined for XNBR_0.4 DCP_2CPO. The absence of measurable adhesion between a silver rod and highly crosslinked NBRs is most likely a physical effect, associated with the different thermal shrinkage of the metal and the rubber after vulcanization.

## 5. Conclusions

The above results demonstrate the potential of using the carboxy-containing peroxy oligomer (CPO) as multifunctional additive for rubber. Its application makes it possible to improve adhesion to metal, promote the peroxide crosslinking of rubber and modify crosslink structure. The use of various amounts of CPO together with dicumyl peroxide (DCP) can modify the properties of rubber vulcanizates to a high extent. 

Adhesion between AgNW and rubbers is important when it comes to polymer nanocomposites filled with silver nanoparticles. Rather surprisingly, there is not much information about silver–polymer interfaces in the subject literature [1]. Apart from silver oxides, present on the surface of silver wires, hydroxyl groups are also potentially reactive towards epoxides. However, in the macroscale, silver hydroxide is unstable due to the favorable energetics for the oxide formation. Nevertheless, some authors claim the presence of hydroxyl groups on the surface of oxidized silver [16], that could react with carboxylic groups. The application of the CPO oligomer can help in the improvement of polymer–filler interactions. It is also worth noting that the resins, in addition to improving interphase interactions, can also contribute to crosslinking of rubber matrix by the increasing of crosslinks density and possibly modifying their structure. Increased content of longer, more elastic or even labile crosslinks reduces interfacial tension [19]. Such modification could be beneficial in terms of mechanical properties, adhesion and eventually the durability of rubber sensors containing silver nanowires, also influencing their conducting behavior, to which has the most attention been paid so far [20].

To sum up, the observed adhesion between the rubbers studied and a silver rod is the result of several factors, such as:

1. The presence of sterically available functional groups that can form strong covalent or covalent–ion metal–polymer bonds, additionally introduced by the modification of the rubber by the CPO resin;
2. The optimal degree of rubber crosslinking, also modified by the resin, ensuring the coherence of the polymer matrix; and
3. The conditions of vulcanization and the processing of materials which can introduce stresses at the metal–polymer interface.

SBR crosslinked by DCP with the addition of CPO or XNBR, but providing that 0.4 phr of DCP + 0.2 phr of CPO system is used, can be recommended as potential matrices for nanocomposites filled with silver nanowires, serving e.g., as strain sensors. However, the general conclusion is that a small addition, within ca. 0.2 phr of oligomeric peroxide containing polar groups, for selected SBR and XNBR rubbers, may improve their interactions with silver particles or adhesion to their surface, justifying further research in this direction.

## Figures and Tables

**Table 1:** Composition of the rubber compounds studied (phr).

Compound	Rubber	DCP	CPO
SBR_0.2DCP	100	0.2	0
SBR_2CPO	100	0	2
SBR_0.2DCP_2CPO	100	0.2	2
SBR_0.4DCP	100	0.4	0
SBR_0.4DCP_2CPO	100	0.4	2
NBR_0.2DCP	100	0.2	0
NBR_2CPO	100	0	2
NBR_0.2DCP_2CPO	100	0.2	2
NBR_0.4DCP	100	0.4	0
NBR_0.4DCP_2CPO	100	0.4	2
XNBR_0.2DCP	100	0.2	0
XNBR_2CPO	100	0	2
XNBR_0.2DCP_2CPO	100	0.2	2
XNBR_0.4DCP	100	0.4	0
XNBR_0.4DCP_2CPO	100	0.4	2

Two phr of carboxy-containing peroxy oligomer (CPO) with its peroxide content is equivalent to roughly 0.2 phr of DCP (dicumyl peroxide—98%, Merck (Darmstadt, Germany)), based on the weight content of peroxide groups present in both chemical reagents. SBR—styrene–butadiene rubber: styrene content 23.5%, Ker 1502, Synthos (Oswiecim, Poland); NBR—acrylonitrile–butadiene rubber: acrylonitrile content 17%, Perbunan 1846, Arlanxeo (Dormagen, Germany); XNBR—carboxylated acrylonitrile–butadiene rubber: acrylonitrile content 27%, carboxylic acid content 7%, Krynac X 750, Arlanxeo (Dormagen, Germany).

**Table 2:** Curing parameters of the rubber compounds studied.

Rubber Mix	t_05_ (min)	t_90_ (min)	MH (dNm)	ML (dNM)	ΔM (dNm)
SBR_0.2DCP	1.0	17	5.2	1.0	4.2
SBR_2CPO	0.5	18	2.4	1.0	1.4
SBR_0.2DCP_2CPO	1.0	20	6.8	1.0	5.8
SBR_0.4DCP	1.0	17	10.2	1.0	9.2
SBR_0.4DCP_2CPO	1.0	18	8.7	1.0	7.7
NBR_0.2DCP	1.0	25	6.5	1.4	5.1
NBR_2CPO	1.0	32	3.4	1.4	2.0
NBR_0.2DCP_2CPO	1.0	24	7.5	1.4	6.1
NBR_0.4DCP	1.0	21	9.1	1.3	7.8
NBR_0.4DCP_2CPO	1.0	22	9.2	1.3	7.9
XNBR_0.2DCP	1.0	25	2.4	0.5	1.9
XNBR_2CPO	1.0	40	1.4	0.5	0.9
XNBR_0.2DCP_2CPO	1.2	25	2.4	0.5	1.9
XNBR_0.4DCP	1.0	21	4.7	0.5	4.2
XNBR_0.4DCP_2CPO	1.1	21	4.4	0.5	3.9

t_05_, t_90_—scorch and vulcanization time, respectively, MH and ML—max. and min. torque adequately, ΔM = MH − ML – torque increase.

**Table 3:** Crosslink density and structure of the vulcanizates studied.

Sample	ν (mol/cm^3^)	ν_A_ (mol/cm^3^)	ν − ν_A_ (mol/cm^3^)
SBR_0.2DCP	0.41 × 10^−4^	0.41 × 10−4	0.00
SBR_2CPO	0.00	0.00	0.00
SBR_0.2DCP_2CPO	1.25 × 10^−4^	1.19 × 10^−4^	0.06 × 10^−4^
SBR_0.4DCP_2CPO	0.92 × 10^−4^	0.89 × 10^−4^	0.03 × 10^−4^
SBR_0.4DCP	0.91 × 10^−4^	0.91 × 10^−4^	0.00
NBR_0.2DCP	0.62 × 10^−4^	0.62 × 10^−4^	0.00
NBR_2CPO	1.85 × 10^−5^	0.91 × 10−5	0.09 × 10^−4^
NBR_0.2DCP_2CPO	0.99 × 10^−4^	0.93 × 10^−4^	0.06 × 10^−4^
NBR_0.4DCP_2CPO	1,20 × 10^−4^	1,04 × 10^−4^	0.16 × 10^−4^
NBR_0.4DCP	1,43 × 10^−4^	1,43 × 10^−4^	0.00
XNBR_0.2DCP	1.07 × 10^−4^	1.03 × 10^−4^	0.04 × 10^−4^
XNBR_2CPO	1.04 × 10^−4^	0.65 × 10^−4^	0.39 × 10^−4^
XNBR_0.2DCP_2CPO	1.33 × 10^−4^	1.06 × 10^−4^	0.27 × 10^−4^
XNBR_0.4DCP_2CPO	2.41 × 10^−4^	2.16 × 10^−4^	0.25 × 10^−4^
XNBR_0.4DCP	2.78 × 10^−4^	2.76 × 10^−4^	0.02 × 10^−4^

**Table 4:** Tensile strength and relative elongation at break of the rubber samples tested.

Sample	E_100_ (MPa)	E_200_ (MPa)	E_300_ (MPa)	Tensile Strength (MPa)	Elongation at Break (%)
SBR_0.2DCP	0.57 ± 0.11	0.96 ± 0.08	1.16 ± 0.06	1.59 ± 0.21	510 ± 89
SBR_2CPO	0.40 ± 0.04	0.43 ± 0.04	0.45 ± 0.05	0.50 ± 0.05	595 ± 165
SBR_0.2DCP_2CPO	0.78 ± 0.20	1.14 ± 0.29	1.62 ± 0.49	2.43 ± 0.90	374 ± 81
SBR_0.4DCP	0.72 ± 0.08	1.16 ± 0.00	1.43 ± 0.08	1.51 ± 0.16	313 ± 14
SBR_0.4DCP_2CPO	0.74 ± 0.07	1.31 ± 0.24	-	1.65 ± 0.19	201 ± 21
NBR_0.2DCP	0.81 ± 0.07	1.26 ± 0.15	1.55 ± 0.11	1.68 ± 0.12	287 ± 49
NBR_2CPO	0.41 ± 0.01	0.52 ± 0.04	0.61 ± 0.07	1.02 ± 0.11	643 ± 186
NBR_0.2DCP_2CPO	1.19 ± 0.25	-	-	1.74 ± 0.29	179 ± 59
NBR_0.4DCP	1.04 ± 0.14	-	-	1.49 ± 0.26	169 ± 20
NBR_0.4DCP_2CPO	0.96 ± 0.02	-	-	1.24 ± 0.10	157 ± 29
XNBR_0.2DCP	0.67 ± 0.03	0.77 ± 0.03	0.83 ± 0.03	1.86 ± 0.17	1070 ± 76
XNBR_2CPO *	0.55 ± 0.01	0.55 ± 0.01	0.52 ± 0.06	>0.80 ± 0.06	*
XNBR_0.2DCP_2CPO *	0.67 ± 0.04	0.85 ± 0.07	0.99 ± 0.10	>2.19 ± 0.44	*
XNBR_0.4DCP	0.76 ± 0.03	0.92 ± 0.05	1.06 ± 0.08	2.51 ± 0.29	653 ± 82
XNBR_0.4DCP_2CPO	0.74 ± 0.03	0.96 ± 0.05	1.18 ± 0.36	2.05 ± 0.36	529 ± 92

* Samples not broken under max. experimental range of extension (450 mm).

**Table 5:** Tear strength of the rubber samples tested.

Sample	F_max_ (^N^/_mm_)	F_min_ (^N^/_mm_)
SBR_0.2DCP	6.05	5.78
SBR_2CPO	No tearing	
SBR_0.2DCP_2CPO	3.68	2.85
SBR_0.4DCP	3.5	2.3
SBR_0.4DCP_2CPO	3.6	2.41
NBR_0.2DCP	5.96	3.18
NBR_2CPO	No tearing	
NBR_0.2DCP_2CPO	4.08	2.96
NBR_0.4DCP	2.66	1.72
NBR_0.4DCP_2CPO	2.43	1.78
XNBR_0.2DCP	No tearing	
XNBR_2CPO	No tearing	
XNBR_0.2DCP_2CPO	No tearing	
XNBR_0.4DCP	7.71	5.75
XNBR_0.4DCP_2CPO	7.89	6.75

## Data Availability

The raw/processed data required to reproduce these findings cannot be shared at this time due to technical or time limitations.
